# Non-stationary ^13^C metabolic flux analysis of Chinese hamster ovary cells in batch culture using extracellular labeling highlights metabolic reversibility and compartmentation

**DOI:** 10.1186/1752-0509-8-50

**Published:** 2014-04-28

**Authors:** Averina Nicolae, Judith Wahrheit, Janina Bahnemann, An-Ping Zeng, Elmar Heinzle

**Affiliations:** 1 Universität des Saarlandes Technische Biochemie, Campus A 1.5, Saarbrücken D-66123, Germany; 2 Institute of Bioprocess and Biosystems Engineering, Technische Universität Hamburg-Harburg, Denickestr. 15, Hamburg D - 21073, Germany

**Keywords:** Chinese hamster ovary cells, Metabolic flux analysis, Mitochondria, Compartmentation, Mammalian metabolism, CHO, Mammalian cell culture, Metabolic transport, Reversibility

## Abstract

**Background:**

Mapping the intracellular fluxes for established mammalian cell lines becomes increasingly important for scientific and economic reasons. However, this is being hampered by the high complexity of metabolic networks, particularly concerning compartmentation.

**Results:**

Intracellular fluxes of the CHO-K1 cell line central carbon metabolism were successfully determined for a complex network using non-stationary ^13^C metabolic flux analysis. Mass isotopomers of extracellular metabolites were determined using [U-^13^C_6_] glucose as labeled substrate. Metabolic compartmentation and extracellular transport reversibility proved essential to successfully reproduce the dynamics of the labeling patterns. Alanine and pyruvate reversibility changed dynamically even if their net production fluxes remained constant. Cataplerotic fluxes of cytosolic phosphoenolpyruvate carboxykinase and mitochondrial malic enzyme and pyruvate carboxylase were successfully determined. Glycolytic pyruvate channeling to lactate was modeled by including a separate pyruvate pool. In the exponential growth phase, alanine, glycine and glutamate were excreted, and glutamine, aspartate, asparagine and serine were taken up; however, all these amino acids except asparagine were exchanged reversibly with the media. High fluxes were determined in the pentose phosphate pathway and the TCA cycle. The latter was fueled mainly by glucose but also by amino acid catabolism.

**Conclusions:**

The CHO-K1 central metabolism in controlled batch culture proves to be robust. It has the main purpose to ensure fast growth on a mixture of substrates and also to mitigate oxidative stress. It achieves this by using compartmentation to control NADPH and NADH availability and by simultaneous synthesis and catabolism of amino acids.

## Background

Economic importance and ease of cultivation make CHO cells a desirable candidate for metabolic studies in eukaryotic systems. Alongside with being the most important mammalian cell line for producing biopharmaceuticals [1-3], CHO cells are able to grow in suspension cultures using chemically defined media [4], use multiple carbon sources simultaneously and maintain a stable metabolism for long periods in batch cultivations. This has led to a wealth of studies aimed at exploring CHO metabolism. After the decoding of the CHO-K1 cell line genome [5,6] and transcriptome [7], we can expect such studies to increase both in number and complexity. Metabolic flux analysis has been applied for mammalian cells for a long time already but mostly only using metabolite balancing [8]. The labelling of extracellular excreted lactate and CO_2_ has already been used in the past but only at metabolic steady state [8-11]. The use of other labelled metabolites, e.g. amino acids, is more complex because of the often high reversible exchange with the media [8]. Metabolic flux analysis (MFA) of CHO cell cultures evolved from flux balancing analysis [12] to more complex metabolic or isotopomer dynamic models [13]. Newer studies rely on ^13^C-MFA applied by fitting the summed fractional labeling [14] or by fitting steady-state labeling data [15] resulted from using in parallel more labeled substrates for determining the intracellular fluxes at metabolic steady state in different growth phases. However, the labeling patterns of the intracellular metabolites or of amino acids from hydrolyzed proteins that are usually needed for non-stationary ^13^C-MFA are obtained through a tedious methodology [16,17] and are susceptible to errors stemming mostly from the quenching/extraction phase [18,19]. In the absence of metabolite exchange with the media, intracellular labeling would reach steady state relatively fast, in the order of minutes for glycolytic intermediates and few hours for TCA cycle metabolites, as it was determined in *Pichia pastoris*[20]. In mammalian cells, exchange with the extracellular pools [21] delays the intracellular isotopic steady state usually beyond the possibility to maintain metabolic steady state. Due to the large extracellular pools of amino acids, their exchange will transfer the time constant of the extracellular labeling process, which is in the order of days, to the intracellular labeling. One option is to use isotopic non-stationary metabolic flux analysis (Inst-^13^CMFA) applied at short time scales [22], but this approach has the drawback of requiring accurate determination of intracellular concentrations of metabolites [23].

Metabolite and reaction compartmentation is important for a realistic representation of the mammalian cell metabolism, but determining it is more challenging both concerning experimental and modeling procedures, as we have already reviewed in [24]. In the exponential growth phase, a typical culture of CHO is characterized by high uptake rates of glucose and glutamine, the Warburg effect and the exchange of non-essential amino acids with the extracellular media [4,25]. We can expect that by feeding a ^13^C labeled substrate, some of the extracellular metabolites will exhibit labeling patterns that can then be detected using GC-MS. As these metabolites will be enriched in ^13^C dynamically, non-stationary ^13^C metabolic flux analysis (Inst-^13^CMFA) applied to extracellular and intracellular isotopomers [26-28] provides a suitable framework to determine the intracellular fluxes. Extracellular pools have a large time scale for labeling (hours) compared to the intracellular pools (seconds/minutes), thus removing the need to sample intracellular pools provided that the labeling information in the extracellular metabolites is sufficient.

We show that by using only the labeling patterns of extracellular metabolites produced by feeding [U-^13^C_6_]glucose as the only labeled substrate, intracellular fluxes can be successfully determined in a complex, compartmented metabolic network of the CHO-K1 cell line. In parallel, we prove that a simplified, non-compartmented model is not sufficient for describing the metabolism. We also underline the importance of considering reversibility when dealing with non-stationary isotopomer models.

## Methods

### Cell culture and experimental set-up

The CHO-K1 cell line was kindly provided by the Institute of Cell Culture Technology (University Bielefeld, AG Noll, Germany). The cells were growing in suspension under serum and protein free conditions in the chemically defined medium TC-42 (TeutoCell AG, Bielefeld, Germany) supplemented with 4 mM L-glutamine (PAA, Germany). Precultures were cultivated in 125 mL baffled Erlenmeyer flasks (Corning Inc., Germany) at an initial cell density of 0.4×10^6^ cells/mL and a working volume of 50 mL on a shaking device (225 rpm) at 37°C and 5% CO_2_ in a humid atmosphere. For the main cultivation, cells were harvested during the exponential growth phase at a viability of ≥ 98% and resuspended in TC-42 medium with 100% [U-^13^C_6_] glucose (99%, Euriso-Top, Saarbrücken, Germany). The main cultivation was performed in a Vario1000 bioreactor (Medorex e.K., Nörten-Hardenberg, Germany) at batch mode with a starting culture volume of 200 mL. The bioreactor was inoculated at a cell density of 0.4×10^6^ cells/mL. The cultivation temperature was kept constant at 37°C and the impeller (3-blade marine propeller) speed was set to 300 rpm. During the cultivation, the pH value was controlled at 7.2 by gassing with CO_2_ and by using 0.5 M sodium carbonate solution. Dissolved oxygen was maintained at 30% of the saturation concentration. Samples were taken three times a day. Cell density and viability were determined by cell counting using the Trypan blue exclusion method. Supernatants were transferred into fresh tubes and stored at −20°C until further analysis. The average cell diameter was determined using an automated cell counter (Invitrogen, Darmstadt, Germany) in a separate experiment. This experiment was performed in a shaking incubator (2 inches orbit, 185 rpm, 37°C, 5% CO_2_ supply) using 250 mL baffled Erlenmeyer flasks (Corning Inc., Germany), an initial cell density of 0.4×10^6^ cells/mL, a working volume of 100 mL and using the same TC-42 medium (TeutoCell, Bielefeld, Germany) supplemented with 4 mM glutamine. Differences of cell diameters during the cultivation were maximum 5% and not taken into account. Cell volume was computed assuming the cells are spherical using a diameter of 10.6 μm. Glutamine degradation kinetics were determined experimentally in a cell-free setup identical to the one employed for cell volume estimation. The determined glutamine degradation rate constant was *kd*_*GLN*_ = 0.0033 h^−1^.

### Quantification of metabolites

Quantification of glucose, organic acids and amino acids via HPLC was carried out as described previously by Strigun et al. [29].

### Analysis of isotopomer labeling patterns

#### Sample preparation

For determination of labeling patterns of lactate and amino acids, 50 μl of supernatants were lyophilized, resolved in 50 μl N,N-dimethylformamide (0.1% pyridine) and incubated at 80°C for 30 min. 50 μl N-methyl-N-t-butyldimethylsilyl-trifluoro-acetamide (MBDSTFA) was added followed by another incubation at 80°C for 30 min for derivatization of metabolites into corresponding dimethyl-t-butylsilyl derivatives. For determination of the labeling pattern of pyruvate, lyophilized supernatants were resolved in 50 μl pyridine containing 20 mg/ml methoxyamine hydrochloride and 50 μl MSTFA (Macherey-Nagel, Düren, Deutschland) and incubated at 80°C for 30 min for derivatization into the methoxyamine-trimethylsilyl derivative. Derivatized samples were centrifuged at 13000 × g for 5 min at 4°C and supernatants transferred into fresh glass vials with micro inlets.

#### GC-MS measurements

Extracellular ^13^C-labeling dynamics were analyzed by gas chromatography mass spectrometry (GC-MS). The GC-MS measurements were carried out on a GC (HP 6890, Hewlett Packard, Paolo Alto, CA, USA) equipped with an HP5MS capillary column (5% phenyl-methyl-siloxane diphenylpolysiloxane, 30 m × 0.25 mm × 0.25 μm, Agilent Technologies, Waldbronn, Germany), electron impact ionization at 70 eV, and a quadrupole detector (Agilent Technologies). The injection volume was 1 μl (7683B Autosampler, Agilent, Waldbronn, Germany; PTV-Injektor, Gerstel, Mühlheim a. d. Ruhr, Germany). Helium was used as carrier gas at a flow rate of 1.1 ml/min for analysis of lactate and amino acids or 0.7 ml/min for pyruvate analysis. The following temperature gradient was applied for lactate and amino acid analysis: 135°C for 7 min, 10°C/min up to 162°C, 7°C/min up to 170°C, 10°C/min up to 325°C, 325°C for 2.5 min; inlet temperature: 140°C and heating with 720°C/min up to 320°C; interface temperature 320°C; quadrupole temperature 150°C. The temperature gradient for pyruvate analysis was as follows: 70°C for 1 min, 1°C/min up to 75°C, 5°C/min up to 315°C, 25°C/min up to 340°C, 340°C for 5 min; inlet temperature: 70°C and heating with 360°C/min up to 360°C; interface temperature 320°C; quadrupole temperature 280°C.

#### Data analysis

After identification of metabolites in the scan mode using the NIST data bank, quantification of labeling enrichment was done in SIM (single ion monitoring) mode in two technical replicates using the following unique fragments (m/z) containing the complete carbon skeleton of metabolites: pyruvate 174, lactate 261, alanine 260, glycine 246, serine 390, aspartate 418, glutamate 432, glutamine 431. Mass isotopomer distributions were corrected for naturally occurring isotopes using the method of Yang et al. [30].

### Metabolic network models

**Figure 1:**
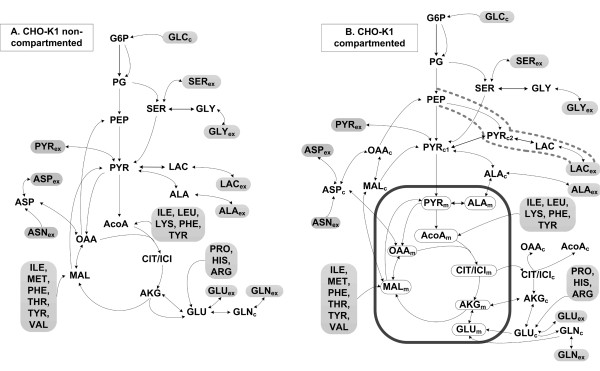
**Non-compratmented (A) and compartmented (B) networks of the CHO-K1 central metabolism used for simulations.** Irreversibility is indicated by simple arrows, and reversibility by double arrows. The reactions depicted in 1B are listed in detail in the Additional file 1 together with fluxes and reversibilities determined. Subscripts meaning: *ex –* extracellular*; c –* cytosolic*; m –* mitochondrial*.* Abbreviations: AA –amino acids; AcoA – acetyl CoA; AKG – alpha-ketoglutarate; ALA – alanine; ASN – asparagine; ASP – aspartate; CIT/ICI – citrate/isocitrate; G6P – glucose 6-phosphate; GLC – glucose; GLN – glutamine; GLU – glutamate; GLY – glycine; MAL – malate; OAA – oxaloacetate; PEP – phosphoenolpyruvate; PG – phosphoglycerate; PYR – pyruvate; SER – serine.

Two metabolic networks were established based on experimental observations related to metabolite uptake and production and extracellular labeling. Both networks included: glycolysis; TCA cycle; anaplerotic reactions; synthesis of fatty acids, proteins and carbohydrates for biomass production; amino acid production and degradation. Transport from the extracellular media was reversible in both models for all metabolites with the exception of glucose, asparagine and essential amino acids. Mitochondrial transport of malate, α-ketoglutarate, alanine, and reactions of transaminase, malate and lactate dehydrogenase were also reversible. The first model shown in Figure 1A considers the intracellular space without compartmentation. In the second model (Figure 1B), the mitochondrial reactions and pools are separated from the cytosol. Both models start from the annotation of the genomes of CHO-K1 and *Mus musculus*[31-33]. Enzyme localization was established using information from the MGI database and data from J. Wahrheit [34] who measured compartmented enzyme activity using a method adapted from Niklas et al. [35]. Mitochondrial transport of alanine was included to explain the existence of alanine aminotransferases in both compartments. Metabolite pools were lumped where it did not influence the simulated labeling dynamics. Pentose phosphate pathway was reduced to one reaction where one carbon atom is lost for each G6P molecule and 5/3 molecules of PG are produced. Glycolysis was lumped to three fluxes transforming G6P into PYR_cyt_. Isocitrate and citrate were condensed into one citrate (CIT) pool. Succinate, fumarate and malate were condensed into one MAL pool. Two cytosolic pyruvate pools were used to describe metabolic channeling to lactate. Non-essential amino acids catabolism was lumped to three fluxes fueling the MAL, AcoA and GLU pool respectively. No carbon mapping was required in this case as essential amino acids are unlabeled. Glutaminase activity was mitochondrial [5] and glutamine synthetase was cytosolic [36]. Fatty acids, protein and storage carbohydrates composition of the cell was taken from Altamirano et al. [12].

In total, the compartmented model consisted of 60 fluxes and 25 metabolites and the non-compartmented model of 42 fluxes and 16 metabolites. The complete flux list for the two models, together with the carbon transfer rules, is provided in the Additional file 1.

### Non-stationary-^13^CMFA methodology

Isotopic non-stationary metabolic flux analysis (Inst-^13^CMFA) comprises: (1) metabolic steady-state balancing of intracellular metabolites for determining extracellular rates; (2) dynamic extracellular metabolite and isotopomer balance and (3) dynamic balances of intra-compartmental isotopomers.

### Metabolite balancing

Net extracellular rates *v*_*M_ex*_ were determined for each extracellular metabolite *M_ex* for the batch cultivation situation, under the assumption of metabolic steady state, by fitting the cell density *X(t)* and extracellular concentrations of metabolites *C*_*M_ex*_ to an exponential growth model with specific growth rate *μ* (eq. 1.a and eq. 1.b) and constant extracellular rates. Glutamine balance included first order degradation in the culture media (eq. 1.c).

(1.a) $$\mathrm{dX}/\mathrm{dt}=\mathrm{\mu X}\left(t\right)$$

(1.b) $$dC_{M\_\mathrm{ex}}/\mathrm{dt}=v_{M\_\mathrm{ex}}X\left(t\right)$$

(1.c) $$dC_{\mathrm{GLN}}/\mathrm{dt}=v_{\mathrm{GLN}}\cdot X\left(t\right)-kd_{\mathrm{GLN}}\cdot C_{\mathrm{GLN}}\left(t\right)$$

At intracellular metabolic steady state, the *n* metabolic fluxes that connect the *m* metabolites are constant and satisfy the material balance:

(2.a) G·v=0

(2.b) $$v_{j}=\varphi_{j},j=1..R_{\mathrm{meas}}$$

(2.c) $$\alpha_{i}\leq v_{i}\leq \beta_{i,i=1..n}$$

where *G* is the *m* × *n* stoichiometric matrix and its null space *v* is the vector of net metabolic fluxes which are constrained by *R*_*meas*_ measured fluxes *φ*_*j*_ (2.b) and *n* inequalities (2.c) determined by flux direction. To reduce the number of parameters, the free fluxes were extracted from the network as described in [37] to produce a determined stoichiometric system.

### Intracellular and extracellular carbon balance

The Inst- ^13^CMFA framework developed in [23,28] was adapted to the case of batch culture cultivation. Isotopomer balances for extracellular (eq. 3) and intracellular (eq. 4) metabolites were set to be solved together with the extracellular balances (eq. 1.a, 1.b, 1.c).

(3) $$\begin{array}{l} \frac{\mathrm{dID}V_{M\_\mathrm{ex}}}{\mathrm{dt}}=\frac{1}{C_{M\_\mathrm{ex}}}\cdot [X_{\mathrm{conc}}\cdot \left(v_{M\_\mathrm{ex}}^{\mathrm{in}}\cdot \mathrm{ID}V_{M\_\mathrm{cyt}}-v_{M\_\mathrm{ex}}^{\mathrm{out}}\cdot \mathrm{ID}V_{M\_\mathrm{ex}}\right)\\ \;-\frac{dC_{M\_\mathrm{ex}}}{\mathrm{dt}}\cdot \mathrm{ID}V_{M\_\mathrm{ex}}] \end{array}$$

(4) $$\frac{\mathrm{dID}V_{M\_\mathrm{in}}}{\mathrm{dt}}=\frac{1}{C_{M\_\mathrm{in}}}\cdot \left(\sum_{j=1}^{R_{M}}v_{j}\cdot \mathrm{ID}V_{M\_\mathrm{in},j}-v_{M\_\mathrm{in}}^{\mathrm{out}}\cdot \mathrm{ID}V_{M\_\mathrm{in}}\right)$$

with *IDV*_*M_ex*_*, IDV*_*M_cyt*_*, IDV*_*M_in*_*,* being the isotopomer distribution vectors of the extracellular, cytosolic and intracompartmental pools of metabolite *M. IDV*_*M_in,j*_ is the *j*^*th*^ reaction contribution to isotopomers of metabolite *M,* computed using isotopomer mapping matrices as described by Schmidt [28]. *X*_*conc*_ is the cell volumetric concentration expressed in L cell/L media. *C*_*M_ex*_ is the extracellular concentration of *M*, $v_{M\_\mathrm{ex}}^{\mathrm{in}}$ is the production flux of *M* expressed in mmol/(L cell × h), $v_{M\_\mathrm{ex}}^{\mathrm{out}}$ is the uptake flux, *v*_*j*_ is one of the *R*_*M*_ fluxes entering the intracompartmental pool of *M, C*_*M_in,*_ and $v_{M\_\mathrm{in}}^{\mathrm{out}}$ is the flux exiting the pool. The metabolite and isotopomer balances from equations 1,2 and 4 are then solved simultaneously to obtain the time course of the mass isotopomer distributions. Isotopomer balancing employs absolute fluxes that can be computed from the net fluxes by introducing a reversibility parameter:

(5) $$\mathrm{re}v_{j}=\frac{v_{j}{{}_{,}}_{\mathrm{reverse}}}{v_{j}}$$

where *v*_*j*_ is the net flux and *v*_*j, forward*_ and *v*_*j, reverse*_ are the forward and respectively the reverse exchange fluxes, with *v*_*j, forward*_ – *v*_*j,reverse*_ *= v*_*j*_; *v*_*j, forward*_*≥0* and *v*_*j,reverse*_*≥0*.

The contribution of reaction *j* was computed using isotopomer mapping matrices [28] that trace carbon from the substrate to the reaction products. The initial mass distribution of all metabolites was computed considering the naturally occurring ^13^C fraction (1.1%) and the 99% atom purity of the employed ^13^C labeled substrate.

The simulated time course of extracellular mass isotopomer distributions (*MID*) is compared with the experimental values. The objective function to be minimized was expressed as the weighted sum of square differences between the experimentally determined and simulated MIDs:

(6) $$\mathrm{SSQD}={\left(\mathrm{MI}D^{\mathrm{sim}}-\mathrm{MI}D^{\text{exp}}\right)}^{T}\cdot \sum_{\mathrm{MID}}^{-1}\cdot \left(\mathrm{MI}D^{\mathrm{sim}}-\mathrm{MI}D^{\text{exp}}\right)$$

where *SSQD* is the objective function, *MID*^*sim*^ is the simulated *MID*, *MID*^*exp*^ is the measured *MID* and *Σ*_*MID*_ is the measurement covariance matrix. The optimal solution was accepted if it satisfied the χ-squared test for model verification with 95% probability, and *N-p* degrees of freedom, where *N* is the number of sampled points (size of *MID*^*exp*^) and *p* is the number of free parameters. To reduce the bias in the objective function generated by very small standard deviations, a minimum threshold of 0.005 was imposed. Accurate confidence intervals and sensitivity analysis of fluxes were computed according to [38]. All the code was programmed and simulated in Matlab [MATLAB and Simulink Release 2013a, The MathWorks, Inc., Natick, Massachusetts, United States].

## Results and discussion

### Metabolic profiling

**Figure 2:**
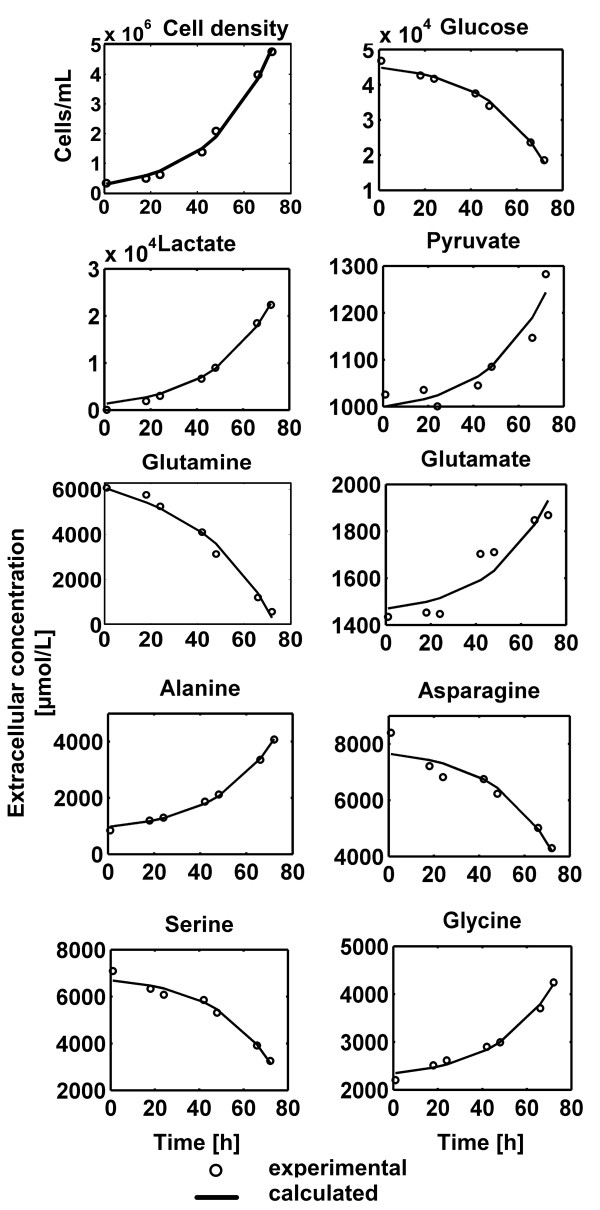
**Culture profile of the CHO-K1 cells for the first 72 h during the exponential growth phase.** Experimental values are shown with circles and calculated values are represented by solid lines.

The cells exhibited exponential growth for 72 h (Figure 2) until glutamine became exhausted and a shift in metabolism was observed (data not shown). Estimated specific growth rate as fitted to eq. (1.a) was *μ* = 0.0401 h^−1^. Uptake and production of most metabolites was balanced, i.e. they were proportional to growth for the main carbon sources and produced metabolites (Figure 2) and for other amino acids (Additional file 2).This means that metabolic steady state was maintained during the first 72 h of cultivation. Glucose constituted the main carbon source (Table 1), providing 65% of the total carbon entering the central carbon metabolism, with an uptake flux of 371 mmol/(L cell × h). Note that all fluxes are related to the cell volume specified by L cell. 39% of the glucose was converted to lactate. The observed pyruvate production rate was 3.3 mmol/(L cell × h).

**Table 1:** Carbon sources for the central metabolism of the CHO-K1 cell line in batch culture during the exponential growth phase

**Metabolite**	**Target intracellular metabolite**	**Uptake flux [mmol/(L cell × h)]**	**Uptake flux [Cmmol/(L cell × h)]**	**Percentage of the total carbon-flux**
Glucose	Pyruvate	371.0	2226.2	64.9
Glutamine	AKG	66.4	331.9	9.7
AA1^*^	AcoA	92.6	185.2	5.4
AA2^*^	Malate	49.6	198.4	5.8
AA3^*^	AKG	11.6	58.2	1.7
ASP/ASN	OAA	68.1	272.3	7.9
Serine	Pyruvate, glycine	48.3	146.4	4.3
**TOTAL**	**-**	**-**	**3428.53**	**100**

The contribution of AA1, AA2 and AA3 amino acid groups considers only catabolism. Abbreviations: AA1: amino acids catabolized to AcoA (isoleucine, leucine, lysine, phenylalanine, tyrosine); AA2: amino acids catabolized to four-carbon dicarboxylic acids (isoleucine, methionine, phenylalanine, threonine, tyrosine, valine); AA3: amino acids catabolized to glutamate (arginine, histidine, proline); AcoA: acetyl coenzyme A; AKG: alpha-ketoglutarate; ASN: asparagine; ASP: aspartate; OAA: oxaloacetate.

*Excluding requirements for protein synthesis.

The glutamine uptake flux determined by fitting eq. (1.c) to the glutamine concentration over time was 66.4 mmol/(L cell × h), 16% smaller than that in the case when its degradation was ignored. Glutamine uptake contributed with 10% to the total carbon pool. The rest of the carbon feeding the central carbon metabolism, i.e. 25%, was obtained from amino acids catabolism.

**Figure 3:**
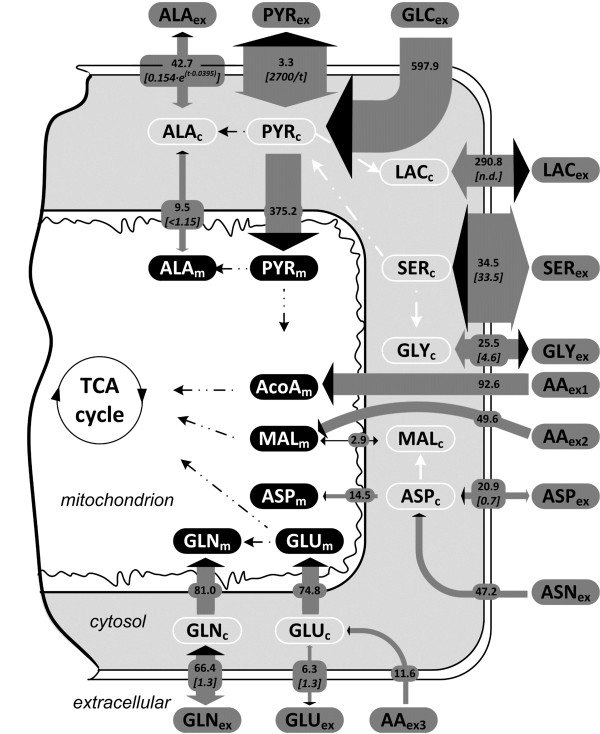
**Compartmentation of the CHO-K1 metabolism and the fate of extracellular metabolites.** Net fluxes are indicated on the gray arrows in units of mmol product/ (L cell × h), and reversibility parameter defined as *reverse flux/net flux* is shown in the square brackets (n.d. = not determined). The thickness of the gray arrows is proportional to the forward flux (=*reverse flux + net flux*), and shown qualitatively for the fluxes with variable reversibility. Net flux direction is shown by the black arrow heads. Amino acids catabolism is represented as the sum of the differences between amino acid uptake flux and flux required for protein production, reported to the metabolite derived from catabolism. Subscripts meaning: *ex –* extracellular*; c –* cytosolic*; m –* mitochondrial*.* Abbreviations: AA_ex1_ – isoleucine, leucine, lysine, phenylalanine, tyrosine catabolized to acetyl-CoA; AA_ex2_ - isoleucine, methionine, phenylalanine, threonine, tyrosine, valine catabolized to fumarate and succinate; AA_ex3_ – arginine, histidine, proline catabolized to glutamate; AcoA – acetyl CoA; ALA – alanine; ASN – asparagine; ASP – aspartate; CIT/ICI – citrate/isocitrate; GLN – glutamine; GLU – glutamate; GLY – glycine; MAL – malate; PYR – pyruvate; SER – serine.

Alanine, glycine and glutamate were produced (Figure 3), while the other amino acids were taken up in excess of the quantity required for biomass synthesis. As a consequence of amino acids catabolism, a flux of 92.6 mmol/(L cell × h) fueled the mitochondrial acetyl-CoA pool from the degradation of isoleucine, leucine, lysine, phenylalanine and tyrosine, while a flux of 39.1 mmol/(L cell × h) cytosolic AcoA was directed towards fatty acids synthesis. The catabolism of excess isoleucine, methionine, phenylalanine, threonine, tyrosine and valine that remained after protein synthesis produced 49.6 mmol/(L cell × h) succinate and fumarate (lumped into one pool of four carbon di-carboxylic acids, here represented as MAL).

Metabolite dilution by growth was neglected due to negligible influence on the total mass balance.

### Non-stationary labeling experiment

**Figure 4:**
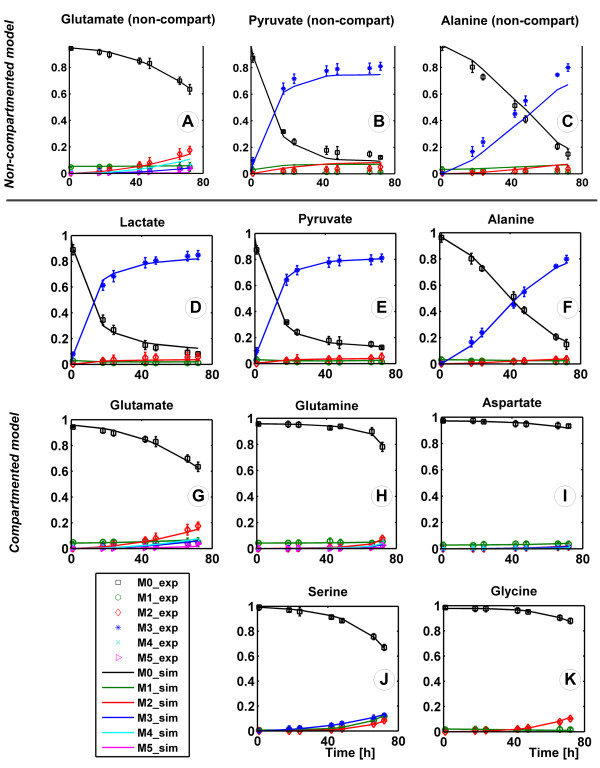
**Non-stationary **^**13**^**C labeling experiment.** Experimental mass isotopomer distributions (symbols) with their standard deviations vs. simulated (line) mass isotopomer distributions of labeled extracellular metabolites. The plots A-C represent results from using the non-compartmented metabolic network specified in Figure 1A. For the other eight plots (D-K), the compartmented model provided in Figure 1B was simulated.

The *MID* of extracellular pyruvate, lactate, alanine, glutamate, glutamine, aspartate, serine and glycine was sampled at 1, 18, 24, 42, 48, 66 and 72 h. During the exponential growth phase of 72 h none of the labeling reached steady state as shown in Figure 4. Lactate and pyruvate exhibited similar labeling dynamics, however with different *MIDs* towards the end of the growth. This is surprising since lactate is obtained from pyruvate through the lactate dehydrogenase reaction. The predominant lactate fraction, i.e. M + 3, increased to 0.85 and the pyruvate M + 3 fraction stabilized at 0.81, pointing towards glycolytic channeling to lactate achieved by the localized cooperation of glycolytic enzymes as observed in rapidly proliferating cells [39,40]. From the produced amino acids, alanine, also derived from pyruvate, had a high M + 3 fraction. Glutamate and glycine M + 2 fractions increased slowly, with most of the change happening in the last 24 h due to the high number of producing cells present in the media., Extracellular aspartate, glutamine and serine were found to be labeled although they exhibited a net uptake. Glutamine fractional labeling, mostly the M + 2 isotopomer, increased sharply at the end of the phase, when very little glutamine remained in the media and the contribution of secreted glutamine played a large role to the labeling state.

### Isotopomer fitting

Both the non-compartmented and compartmented isotopomer network models (Figure 1) were fitted to the experimental mass distributions with the goal of determining unknown fluxes and reversibilities. The 7 sampling time points of the 8 metabolites produced a total number of 252 experimental *MIDs*.

Convergence to the optimal solution is difficult in isotopomer models [41] and the parameter space of the objective function is marked by a multitude of local minima [42], making gradient-based algorithms unreliable. Consequently, we applied a global optimization scheme that had the following steps: (1) generate an initial random population of (*40* x *p*) parameter sets that satisfy constraints using a simulated annealing-based algorithm, (2) submit the population to a *50*-generations genetic algorithm optimization, and (3) refine the best solution using a trust region reflective algorithm. Convergence to the optimal solution was verified by repeating the optimization scheme. One simulation took about 3 s, and the optimization procedure required about 40 h on a 2.3 GHz QuadCore CPU. All the numerical integration and optimization algorithms are found in Matlab toolboxes.

We had initial difficulties in fitting pyruvate and alanine labeling dynamics. As it was shown that reversibility greatly affects labeling dynamics [43], we assumed that the transport reversibility parameter changes in time, even if the net fluxes remain constant. The decrease with time of pyruvate transport reversibility was mechanistically expressed using a hyperbolic function $\mathrm{re}v_{\mathrm{PYR}}=\frac{\mathrm{re}v_{\mathrm{PYR}}^{0}}{\mathrm{time}+\epsilon }$, where pyruvate transport reversibility *rev*_*PYR*_ decreases from a starting value $\mathrm{re}v_{\mathrm{PYR}}^{0}$. To avoid division by zero, a negligible correction factor *ϵ* was introduced. Alanine transport into the cell intensifies as extracellular alanine becomes exponentially more abundant. Transport reversibility was expressed in this case as *rev*_*ALA*_ = *α* · exp(*β* · *time*), where *α* and *β* are parameters to be determined.

The 24 free parameters of the non-compartmented model (Figure 1A) consisted of 5 fluxes, 18 reversibilities and the CO_2_ pool. At convergence, the model failed to fit the data with the minimized *SSQD* of 1572, larger than *χ*^2^ (0.95, 252–24) = 264.2. Pyruvate, lactate, alanine and glutamate labeling were fit poorly even when transport reversibility was variable (Figure 4). In consequence, the non-compartmented model was rejected. The low labeling content of pyruvate, alanine and lactate simulated with the non-compartmented model is explained by the lumping of the cytosolic and mitochondrial pyruvate pools. As more than 30% of the carbon feeding the TCA cycle is not labeled, it is expected that the cataplerotic reactions catalyzed by phosphoenolpyruvate carboxykinase and malic enzyme will produce a large quantity of unlabeled pyruvate, which contradicts the experimental observations.

The compartmented model, consisting of 11 free fluxes and other 27 free parameters (reversibilities) depicted in Figure 1B, fitted the data successfully with the minimized *SSQD* = 249.0 slightly smaller than *χ*^2^ (0.95, 252–38) = 249.13. The complete experimental and simulated datasets, together with the standard deviation of the measured mass isotopomer distributions, are listed in Additional file 3. The poorer fit of the 66 and 72 h time points for lactate and 72 h for alanine can be explained by the metabolic shift towards the end of the growth phase. From a parameter fitting point of view, exponential growth will add a larger contribution in the objective function to the labeling towards the end of the exponential phase compared to the beginning of the experiment because the rates of ^13^C accumulation in the extracellular media will be much larger at high cell densities. This is best evidenced in Figure 4 where glutamine, aspartate, serine and glycine do not become noticeably labeled until 40 h after the introduction of the labeled substrate.

### Metabolic fluxes in the CHO-K1 cell line

**Figure 5:**
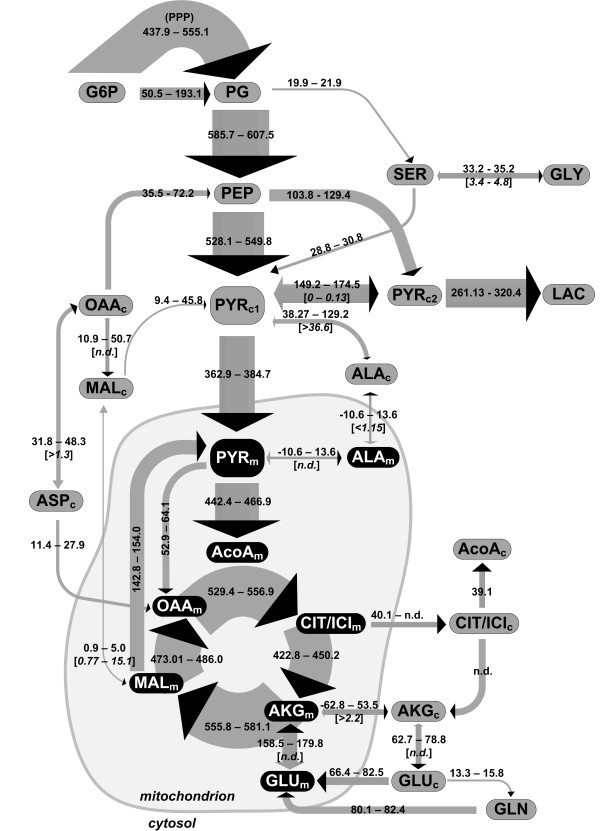
**Estimated net intracellular fluxes in the CHO-K1 central metabolism.** Qualitatively shown by the arrow thickness and their 95% confidence intervals (number interval) together with reversibility confidence intervals (square brackets). *n.d*. – not determined.

Glucose was converted to PG mostly by bypassing glycolysis (Figure 5) through the pentose phosphate pathway (PPP). The estimated PPP flux was 80% of the total molar glucose input flux, a high activity contrasting with results obtained by Ahn and Antoniewicz for adherently growing CHO cells [14] but observed for hybridoma [44] and cancer cells [45]. A wide range of PPP activities, between 0–160% of the glucose input flux, was determined for a highly-productive CHO line in fed-batch cultivation conditions at different growth phases [15]. The large quantities of cytosolic NADPH produced through PPP are used to drive fatty acids synthesis and possibly to mitigate oxidative stress by reducing reactive oxygen species [46-50], as it has also been proposed by [15]. Overflow to lactate comprised 39% of the pyruvate produced from glycolysis. From the rest of the cytosolic pyruvate, 42.7 mmol/(L cell × h) were converted to alanine, but most of it was transported into the mitochondria and converted to AcoA. The channeling flux from PEP to lactate was 122.7 mmol/(L cell × h), accounting for 42% of the total lactate being produced. Low reversibility (Figure 5) meant no connection between the two cytosolic pyruvate pools PYR_c1_ and PYR_c2_ existed. However, lactate was produced from both cytosolic pyruvate pools, indicating that glycolytic channeling is not the only lactate source in the cell. Our possible explanation is that multi-enzyme complexes associated to membrane transporters, as characterized by Campanella et el. [51], create a micro-compartmented environment in the cytosol. Glycolytic enzymes are partly associated and partly soluble, resulting in a mixed response in the lactate labeling.

The carbon flux in the TCA cycle originated mainly from AcoA produced from glycolytic pyruvate transported into the mitochondria (Figure 5), with significant contributions from glutamine and essential amino acids catabolism. Such a high activity of the TCA cycle and high connectivity with the glycolysis is in contrast with some previous reports of lower activity during exponential growth phase [14,52,53] but similar to [54-56]. The differences can be assigned mainly to the use of different cell lines and cultivation conditions like media composition, aeration mode, pH control and culture type e.g. suspension or immobilized, batch or fed-batch. The lower lactate/glucose molar ratio of 0.78 reported herein means more pyruvate is available for use in the TCA cycle, thus making for a more efficient metabolism. Gluconeogenesis was active through PEP carboxykinase with 10% of the total flux entering the PEP pool, a fact explained qualitatively by the presence of M + 2 lactate and pyruvate. In the absence of gluconeogenesis, only the M and M + 3 mass isotopomers of these metabolites would be present after feeding fully labeled glucose. Malic enzyme activity was negligible in the cytosol, and this is in agreement with compartmented enzyme activity observed by J. Wahrheit [34]. This observation reaffirms that PPP was the main source of cytosolic NADPH. Mitochondrial malic enzyme was highly active, producing one third of the total mitochondrial pyruvate. However, a part of the mitochondrial pyruvate was recycled back into the TCA cycle via pyruvate carboxylase. Mitochondrial malate net transport flux was small and reversible. This explained the lack of M + 1 labeling in lactate, alanine and pyruvate that would have been otherwise linked to the M + 2 malate isotopomers that are expected to be obtained in the TCA cycle. As a consequence, the M + 2 labeling in these metabolites relies on mitochondrial transport of citrate and on the activity of citrate lyase producing cytosolic AcoA and OAA, which is then further converted to PEP.

About one third of the total serine was produced from PG, using cytosolic glutamate for transamination. Serine was exchanged with the media, thus explaining extracellular labeling of serine. Half of the serine was not used for protein synthesis but was reversibly converted to glycine and C1 units to sustain the high anabolic activity. Glycine was then secreted. The remaining excess of serine was converted to pyruvate. Alanine was synthesized mainly from cytosolic pyruvate in a highly reversible reaction. Connectivity between cytosolic and mitochondrial alanine pools and the direction of the mitochondrial alanine aminotransferase flux could not be determined. However, the transport flux of alanine to/from mitochondria was confined between −11.6 to 13.6 mmol/(L cell × h), i.e. ±25% of the alanine production flux. The flux of 68.1 mmol/(L cell × h) from asparagine and aspartate uptake to oxaloacetate was split through aspartate aminotransferases between cytosolic and mitochondrial oxaloacetate with a 3/1 ratio, but no other details could be inferred due to the low labeling level in extracellular aspartate.

Isocitrate dehydrogenase (IDH) activity in the cytosol could not be reliably determined due to lack of information in directly connected metabolites citrate and AKG, but also because it affects the labeling pattern in the same way as the mitochondrial isozyme. As a result, the flux in the CIT_m_-CIT_c_-AKG_c_-AKG_m_ cycle could not be determined. Nevertheless, a net activity of cytosolic GDH towards producing the high glutamate flux needed for cytosolic transamination reactions implies that AKG is either produced in the cytosol by IDH or transported from the mitochondria into the cytosol. Mitochondrial glutamate pool was fed by transporting cytosolic glutamate into the mitochondria and by glutamine through GLS activity at comparable rates. In the mitochondria, glutamate was then converted to AKG and fed into the TCA cycle through mitochondrial GDH. In the cytosol, the glutamate produced from AKG in the various transaminase reactions was partially converted to glutamine, which was then exchanged with the media, leading to the presence of labeled glutamine in the media. In conclusion, simultaneous degradation and synthesis pathways for glutamine involve glutamine uptake, transport into the mitochondria and conversion to glutamate, glutamate dehydrogenation to AKG, AKG transport to the cytosol or citrate transport and citrate conversion to AKG through cytosolic IDH activity, conversion of AKG to cytosolic glutamate, and cytosolic glutamine synthesis.

### Transport reversibility

A very important part in modeling the extracellular labeling was considering the reversible exchange between the intracellular pools and the extracellular media, a phenomenon which affects the dynamics of the labeling process. All sampled extracellular non-essential amino acids except asparagine and proline, either produced or taken up, were exchanged with the culture media (Figure 3). Even if the production flux of alanine remained constant throughout the cultivation, the fitting remained poor for alanine when considering a constant reversibility factor. There, the reversibility was estimated to increase with time. The function *rev*_*ALA*_ = 0.154 · exp(0.0359 · *time*) was used to compute the forward and reverse exchange fluxes (eq. 5), with both parameters being determined with a narrow confidence interval (Additional file 1). Time is computed in hours. This successfully explained the dynamics of alanine labeling. The time constant of the reversibility function is a value close to the specific growth rate, pointing to the fact that alanine re-uptake is correlated to the extracellular concentration. Serine secretion flux, as computed with eq. 5, was up to 35 times higher than the net uptake flux. Glycine re-uptake flux was 4.6 times the net production flux. Aspartate, glutamate and glutamine exchange fluxes were in the same order with the net uptake/production flux, as expressed by the estimated reversibility parameter values of about 1. The confidence intervals for the transport reversibility parameters are larger than those for fluxes because at high reversibilities the labeling becomes less sensitive to small changes in reversibility.

Pyruvate transport reversibility is described by the function $\mathrm{re}v_{\mathrm{PYR}}=\frac{2700}{\mathrm{time}+0.01}$, where *time* is specified in hours. The hyperbolic function implies that at the beginning of the cultivation, the intense exchange of pyruvate [57] eliminates the difference between the labeling of the intracellular and extracellular pools. Pyruvate re-uptake decreases because pyruvate concentrations changes slightly (Figure 1) while lactate accumulates in the media to reach high concentrations and competes with pyruvate for the monocarboxylate transporters [58,59]. Lactate transport reversibility parameter could not be estimated because at the beginning of the cultivation there is no lactate present in the media that could dilute the intracellular pool and affect the labeling dynamics.

### Confidence intervals calculation and sensitivity analysis

Most of the fluxes depicted in Figure 5 were determined with narrow confidence intervals. Interval boundaries are not symmetrical due to the non-linear characteristics of the mathematical model. Determining both the flux and exchange in alternative pathways was not possible in the case of high reversibility e.g. for determining the compartmentation of alanine metabolism involving reversible transaminase reactions.

**Figure 6:**
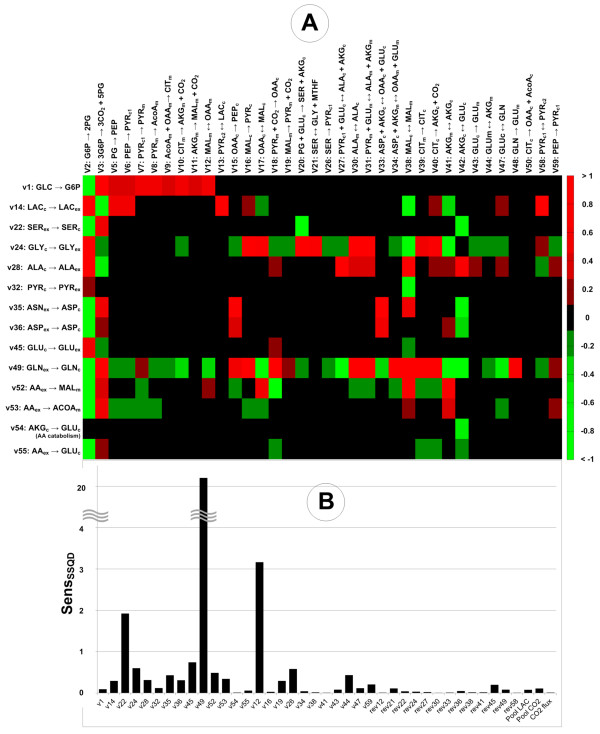
**Sensitivity analysis to measured fluxes and to parameters.** The sensitivity represented in the heat map **(A)** was computed for the compartmented network of CHO-K1 when [U-^13^C_6_] glucose was used as a labeled substrate, $\mathrm{Sen}s_{v_{m}}^{v_{e}}=(dv_{e}/v_{e}^{*})/(dv_{m}/v_{m}^{*})$, where $\mathrm{Sen}s_{v_{m}}^{v_{e}}$ is the sensitivity of the estimated flux *v*_*e*_, re-estimated using values of the measured flux *v*_*m*_ at the border of the confidence interval. *v*_m_^*^ is the average measured flux. The bar chart below **(B)** shows the normalized sensitivity of the objective function (*SensSSQD*) to the free parameters (fluxes and rev = reversibilities). The sensitivity was obtained as a mean value of 100 perturbations of each parameter around the estimated value: $\mathrm{SensSSQD}=(\mathrm{dSSQD}/\mathrm{SSQ}D^{*})/(dp_{i}/p_{i}^{*})$, where SSQD* is the optimized value of the objective function (eq. 6), and *p*_i_* is the estimated value of parameter *i*. The rates *v*_*i*_ correspond to the rates in the network shown in Figure 1A. Abbreviations: subscripts: c: cytosolic, ex: extracellular, m: mitochondrial; ALA: alanine; AcoA: acetyl coenzyme A; AKG: alpha-ketoglutarate; ASN: asparagine; ASP: aspartate; CIT: citrate; G6P: glucose 6-phosphate; GLC: glucose; GLN: glutamine; GLU: glutamate; GLY: glycine; LAC: lactate; MAL: malate; OAA: oxaloacetate; PEP: phosphoenolpyruvate; PG: phosphoglycerate; PYR: pyruvate; SER: serine.

The sensitivity coefficients provided quantitative information about the impact of the measured fluxes on the estimated flux values (Figure 6A). Sensitivity analysis also evidenced correlations between external fluxes and network pathways when [U-^13^C_6_] glucose is used. In this case, the *MIDs* of metabolites will depend on the interplay between the multitude of non-labeled carbon sources and the glucose feed, as opposed to organisms that use only one carbon source [60]. The determination of anaplerotic fluxes relied on the supply of four carbon metabolites from amino acids catabolism. Changes in the glutamine uptake flux (Figure 6A) affected most fluxes to a large extent. Nevertheless, the high influence was mainly computational, as any increase of the flux caused depletion of glutamine at the end of the cultivation and dramatically different labeling patterns. Glucose uptake flux affected the estimation of the PPP and TCA cycle fluxes. Errors in measuring glucose concentration over time will propagate in the values of these fluxes, as the glucose uptake flux determines the fraction of ^13^C entering the cell. G6P loses one ^13^C through oxPPP, therefore estimating the split between glycolysis and oxPPP depends highly on determining correctly all carbon sources. This explains the high sensitivity of the glycolysis/oxPPP split to all extracellular fluxes. Unexpected correlations were observed for the glycine production flux that influenced most anaplerotic and aminotransferase fluxes. Glycine is produced at the expense of serine, which is in turn produced from 3-phosphoglycerate, also converting glutamate to alpha-ketoglutarate during transamination, and simultaneously converted to pyruvate, thus affecting the availability of both cytosolic pyruvate and glutamate.

Local sensitivity of the *SSQD* to free parameters computed as the normalized mean deviation of the objective function to variations in the estimated parameters shown in Figure 6B evidenced the determinable parameters and the redundant parameters. Notoriously difficult to determine anaplerotic fluxes PEP carboxykinase and mitochondrial malic enzyme induced a noticeable sensitivity in the objective function. The increased network connectivity, obtained by coupling ALA or ASP deamination to conversion of AKG to GLU, contributed to this fact. Oppositely, most intracellular reversibilities did not influence the parameter estimation results. This can be easily inferred from the fact that while reaction reversibility affects the dynamics of intracellular isotopomers, it does not mirror in the extracellular labeling apart from the reactions altering the carbon backbone. Also, in the situation where high values of the reversibilities resulted from estimation, local perturbations around these values will not influence the *MIDs*.

## Conclusions

We have shown that intracellular fluxes of the CHO-K1 cell line central carbon metabolism in batch culture can be determined for a complex network by making use solely of the mass isotopomers of extracellular metabolites resulted from feeding [U-^13^C_6_] glucose as the only labeled substrate. To this end, non-stationary ^13^C metabolic flux analysis proved an effective tool for unraveling important details of the CHO-K1 metabolism. Pathway compartmentation, e.g. of anaplerotic reactions and amino acid metabolism had to be considered for describing the mass isotopomer distribution. We reckon that this fact plays an essential role in controlling the availability of NADH and NADPH in mitochondria and cytosol, but also in facilitating amino acid catabolism. A cancer-like high activity of the pentose phosphate pathway produced reducing NADPH partly to counteract the oxidative stress generated by the mitochondrial respiration and partly to fuel fatty acids biosynthesis. Cytosolic pyruvate transport is reversible thousand-fold compared to the net production flux, indicating that although it is not a carbon source, pyruvate creates an extracellular environment [61] most probably by functioning as a balancing system for cytosolic NADH [62]. Considering that metabolite exchange with the media played a very important role in determining the intracellular fluxes, we expect that future ^13^CMFA studies of mammalian cells metabolism will include this essential aspect. Compartmentation controls the simultaneous degradation and production of non-essential amino acids. Most likely, the CHO-K1 cells maintain the exponential growth phase under batch conditions by using a well-connected multi-pool system involving metabolite and reaction compartmentation, exchange with the media and inter-compartment exchange for controlling the metabolite and cofactor pools. Further studies on localizing enzyme and transporter activity together with sampling intra-compartmental concentrations would bring valuable contributions at elucidating the function of such cycling pathways. Accurate enzyme kinetics and thermodynamics [63] in mammalian cells would complement the modeling using Inst-^13^CMFA with information about reaction direction and reversibility. The knowledge gained through Inst-^13^CMFA depicts the CHO-K1 central metabolism as a robust, highly interconnected network that ensures fast growth and mitigates stress generated by reactive oxygen species and the accumulation of lactate in the culture media.

Due to the economic importance of CHO cells, efficient production processes leading to high product quality with minimum effort are of utmost importance. In-depth knowledge about CHO metabolism is expected to provide valuable assistance in identifying targets for metabolic engineering and guiding the design of feeding strategies leading to the development of efficient production processes. Overexpression, silencing or knockout of the specific glycolytic enzymes that associate with channeling glucose to lactate could either be used to study the control of the Warburg effect in cancer cells or for improving glucose utilization. Because glutamine is a limiting substrate, overexpressing glutamine synthetase would enable cells to run a more efficient energy metabolism, with higher fluxes in the TCA cycle. However, as we have shown that compartmentation is important in managing metabolites, mitochondrial transporters are likely to constitute important targets for genetic modifications. Inter-compartmental transport of metabolites is a key factor in connecting the cytosol and the mitochondria energetically and we reckon that modifying the genetic expression of transporters will have significant, perhaps surprising effects on the overall metabolism.

Our proposed methodology of sampling the *MID* only in extracellular metabolites for determining intracellular fluxes using Inst-^13^CMFA has the potential of broader applications, as it circumvents the need to extract intracellular metabolites and it is non-invasive to cells. The information contained in the extracellular mass isotopomers has a higher resolution compared to the summed fractional labeling used previously in [14]. This is sufficient for resolving a complex metabolic network when more metabolites are produced and/or exchanged with the culture media. Therefore, we foresee future applications in the study of mammalian metabolism at physiological and pathological conditions, especially related to compartmentation, as reviewed in [64], and oxidative stress, e.g. in cancer, neurodegenerative disorders and ageing. Knowledge about the metabolism at the compartment level will be essential for identifying therapeutic targets and understanding disease mechanisms. Similarly, the method could be applied to other enzymatic systems or prokaryotic cells where an extended metabolite exchange with the media occurs.

## Abbreviations

c: Cytosolic; ex: Extracellular; m: Mitochondrial; AA: Amino acid; ALA: Alanine; AKG: Alpha-ketoglutarate; ASP: Aspartate; ASN: Asparagine; AcoA: Acetyl-coenzyme A; CHO: Chinese hamster ovary; CIT: Citrate; G6P: Glucose-6-phosphate; GDH: Glutamate dehydrogenase; GLS: Glutaminase; GLU: Glutamate; GLN: Glutamine; ICI: Isocitrate; IDH: Isocitrate dehydrogenase; Inst-13CMFA: Non-stationary ^13^C metabolic flux analysis; LAC: Lactate; MAL: Malate; MID: Mass isotopomer distribution; MTHF: Methyltetrahydrofolate; OAA: Oxaloacetate; oxPPP: Oxidative pentose phosphate pathway; PEP: Phosphoenolpyruvate; PG: Phosphoglycerate; PYR: Pyruvate; SSQD: Sum of square differences; TCA: Tricarboxylic acid.

## Competing interests

The authors declare that they have no competing interests.

## Authors’ contributions

AN performed the modeling, simulations and data analysis. JW designed and performed the experimental data analysis. JW and JB performed the experiments. AN drafted the manuscript. JW, APZ and EH were involved in the study design and provided help with the data analysis and finalizing the manuscript. All authors read and approved the final manuscript.

## Supplementary Material

Additional file 1 **List of reactions in the non-compartmented central carbon metabolism of CHO-K1(Table S1).** List of metabolic reactions, fluxes and reversibilities in the compartmented central carbon metabolism of CHO-K1 **(Table S2).** Carbon transfer rules are provided in the parentheses after each reaction. Reversible reactions are designated by double arrows. Reversibility is computed as the ratio between the reverse flux and the net flux. Click here for file

Additional file 2 **Complete culture profile of CHO-K1 during the exponential growth phase.**The lines represent the fitted concentration profiles to the experimental values (dots) and in the boxes are the determined extracellular rates [mmol/(L cell × h )] together with the 95% confidence intervals. Glutamine uptake was determined by considering a spontaneous degradation rate of 0.0033 h^−1^. The exponential growth phase is shown in the last plot. Click here for file

Additional file 3 Experimental and simulated mass isotopomer distributions of extracellular metabolites and used standard deviations. Click here for file
