# Supplementary material for: Non-stationary 13C metabolic flux analysis of Chinese hamster ovary cells in batch culture using extracellular labeling highlights metabolic reversibility and compartmentation
Source: BMC Syst Biol. 2014 Apr 28;8:50. doi: 10.1186/1752-0509-8-50 (PMC4022241; doi:10.1186/1752-0509-8-50)
Supplement: Additional file 1 — List of reactions in the non-compartmented central carbon metabolism of CHO-K1(Table S1). List of metabolic reactions, fluxes and reversibilities in the compartmented central carbon metabolism of CHO-K1 (Table S2). Carbon transfer rules are provided in the parentheses after each reaction. Reversible reactions are designated by double arrows. Reversibility is computed as the ratio between the reverse flux and the net flux. [file 1752-0509-8-50-S1.pdf]

Table 1. List of reactions of the non-compartmented model of the central carbon metabolism of CHO-K1

Flux	*Reaction*
v1	*GLC_{ex} (abcdef) → G6P (abcdef)*
v2	*G6P (abcdef) → PG (abc) + PG (def)*
v3	*3 G6P (abcdef) → 5 PG (def) + 3 CO_2 (a)*
v4	*G6P (abcdef) → Biomass*
v5	*PG (abc) → PEP (abc)*
v6	*PEP (abc) → PYR (abc)*
v7	*PYR (abc) → AcoA (bc) +CO_2 (a)*
v8	*OAA (abcd) + AcoA (ef) → CIT (efbcda)*
v9	*CIT (abcdef)→ AKG (abcde)+ CO_2 (f)*
v10	*AKG (abcde)→ 0.5 MAL (abcd) + 0.5 MAL (dcba) + CO_2 (e)*
v11	*MAL (abcd) ↔ OAA (abcd)*
v12	*PYR (abc) ↔ LAC (abc)*
v13	*LAC (abc) ↔ LAC_{ex} (abc)*
v14	*OAA (abcd) → PEP (abc) + CO_2 (d)*
v15	*MAL (abcd) → PYR (abc) + CO_2 (d)*
v16	*PYR (abc) + CO_2 (d) → OAA (abcd)*
v17	*PG (abc) → SER (abc)*
v18	*SER (abc) ↔ GLY (ab) + MTHF (c)*
v19	*SER_{ex} (abc) ↔ SER (abc)*
v20	*SER (abc)→ Biomass*
v21	*GLY (abc) ↔ GLY_{ex} (abc)*
v22	*GLY (abc) → Biomass*
v23	*SER (abc) → PYR (abc)*
v24	*PYR (abc) + GLU (defgh) ↔ ALA (abc)+ AKG (defgh)*
v25	*ALA (abc) ↔ ALA_{ex} (abc)*
v26	*ALA (abc)→ Biomass*
v27	*PYR (abc) ↔ PYR_{ex} (abc)*
v28	*ASP (abcd) + AKG (efghi) ↔ OAA (abcd) + GLU (efghi)*
v29	*ASN (abcd) → ASP (abcd)*
v30	*ASP_{ex} (abcd) ↔ ASP (abcd)*
v31	*ASP (abcd) → Biomass*
v32	*AKG (abcde) ↔ GLU (abcde)*
v33	*GLU (abcde) ↔ GLU_{ex} (abcde)*
v34	*GLU (abcde) → Biomass*
v35	*GLN (abcde) → GLU (abcde)*
v36	*GLN_{ex} (abcde) ↔ GLN (abcde)*
v37	*AcoA (ab) → Biomass*
v38	*AA_{ex} (ILE, VAL, MET, THR, TYR, PHE) → MAL (AA catabolism)*
v39	*AA_{ex} (LEU, ILE, LYS, TYR, PHE) → ACOA (AA catabolism)*
v40	*AKG (abcde) → GLU (abcde) (AA catabolism)*
v41	*AA(PRO, HIS, ARG) → GLU (AA catabolism)*
v42	*G6P (abcdef) → carbohydrates*

Table 2. List of reactions of the compartmented model of CHO-K1 central carbon metabolism, including carbon transfer rules, and computed fluxes and reaction reversibilities

Flux	*Reaction*	Flux [mmol/L cell·h]	Lower boundary [mmol/L cell·h]	Upper boundary [mmol/L cell·h]	Reversibility [reverse flux/net flux]	Reversibility lower boundary	Reversibility upper boundary
v1	*GLC_{ex} (abcdef) → G6P (abcdef)*	371.0	326.7	415.3	0	0	0
v2	*G6P (abcdef) → PG (abc) + PG (def)*	65.8	25.2	96.5	0	0	0
v3	*3 G6P (abcdef) → 5 PG (def) + 3 CO_2(a)*	97.5	87.6	111.0	0	0	0
v4	*G6P (abcdef) → Biomass*	3.9	-	-	0	-	-
v5	*PG (abc) → PEP_c (abc)*	597.9	585.7	607.5	0	0	0
v6	*PEP_c (abc) → PYR_{c1} (abc)*	540.3	528.1	549.8	0	0	0
v7	*PYR_{c1} (abc)→ PYR_m (abc)*	375.2	363.0	384.7	0	0	0
v8	*PYR_m (abc) → $AcoA_m$ (bc) +CO_2 (a)*	455.8	442.4	466.9	0	0	0
v9	*OAA_m (abcd) + $AcoA_m$ (ef) → CIT_m (efbcda)*	548.7	529.5	556.9	0	0	0
v10	*CIT_m (abcdef)→ AKG_m (abcde)+ CO_2 (f)*	441.7	422.8	450.2	0	0	0
v11	*AKG_m (abcde)→ 0.5 MAL_m (abcd) + 0.5 MAL_m(dcba) + CO_2 (e)*	571.3	555.8	581.1	0	0	0
v12	*MAL_m (abcd) ↔ OAA_m (abcd)*	474.4	473.0	486.0	14.3	0.16	>100
v13	*PYR_{c2} (abc) ↔ LAC_c (abc)*	290.8	261.1	320.4	15.3	0	>100
v14	*LAC_c (abc) ↔ LAC_{ex} (abc)*	290.8	261.1	320.4	n.d.	n.d.	n.d.
v15	*OAA_c (abcd) → PEP_c (abc) + CO_2 (d)*	65.0	35.5	72.2	0	0	0
v16	*MAL_c (abcd) → PYR_{c1} (abc) + CO_2 (d)*	16.4	9.4	45.8	0	0	0
v17	*OAA_c (abcd) ↔ MAL_c (abcd)*	19.3	10.9	50.7	n.d.	n.d.	n.d.
v18	*PYR_m (abc) + CO_2 (d) → OAA_m (abcd)*	59.6	52.9	64.1	0	0	0
v19	*MAL_m (abcd) → PYR_m (abc) + CO_2 (d)*	149.4	142.8	154.0	0	0	0
v20	*PG (abc) → SER (abc)*	21.2	19.9	21.9	0	0	0
v21	*SER (abc)↔ GLY (ab) + MTHF (c)*	34.5	33.3	35.2	4.03	3.43	4.75
v22	*SER_{ex} (abc) ↔ SER (abc)*	48.3	39.2	57.4	33.5	14.0	98.5
v23	*SER (abc)→ Biomass*	4.9	-	-	0	-	-
v24	*GLY (abc) ↔ GLY_{ex} (abc)*	25.5	22.0	29.0	4.6	2.2	6.9
v25	*GLY (abc) → Biomass*	9.0	-	-	0	-	-
v26	*SER (abc) → PYR_{c1} (abc)*	30.0	28.8	30.8	0	0	0
v27	*PYR_{c1} (abc) + GLU_c (defgh) ↔ ALA_c (abc)+ AKG_c (defgh)*	40.1	38.3	129.2	36.6	17.3	>100
v28	*ALA_c (abc) ↔ ALA_{ex} (abc)*	42.7	39.9	45.5	$0.154 \cdot e^{(t*0.036)}$	0.142 (0.038)	0.169 (0.042)
v29	*ALA_c (abc)→ Biomass*	6.7	-	-	0	-	-
v30	*ALA_m (abc)↔ ALA_c (abc)*	9.5	-10.6	13.6	0.15	0	1.15
v31	*PYR_m (abc) + GLU_m (defgh) ↔ ALA_m (abc)+ AKG_m (defgh)*	9.5	-10.6	13.6	n.d.	-	-
v32	*PYR_{c1} (abc) ↔ PYR_{ex} (abc)*	3.3	2.1	4.56	2700/t	2055/t	2808/t
v33	*ASP_c (abcd) + AKG_c (efghi) ↔ OAA_c (abcd) + GLU_c (efghi)*	45.3	31.9	48.3	19.3	1.3	>100
v34	*ASP_c (abcd) + AKG_m (edghi) → OAA_m (abcd) + GLU_m (efghi)*	14.5	11.4	27.9	0	0	0
v35	*ASN (abcd) → ASP_c (abcd)*	47.2	31.1	63.2	0	0	0
v36	*ASP_{ex} (abcd) ↔ ASP_c (abcd)*	20.9	1.1	40.7	0.7	0.34	0.84
v37	*ASP_c (abcd) → Biomass*	8.4	-	-	0	-	-

v38	*MAL_c (abcd) ↔ MAL_m (abcd)*	2.9	0.9	5.0	6.9	0.8	15.1
v39	*CIT_m (abcdef) → CIT_c (abcdef)*	106.6	40.1	n.d.	0	0	0
v40	*CIT_c (abcdef) → AKG_c (abcde)+ CO_2 (e)*	67.6	1	n.d.	0	0	0
v41	*AKG_m (abcde) ↔ AKG_c (abcde)*	26.3	-62.8	53.5	8.2	2.15	>100
v42	*AKG_c (abcde) ↔ GLU_c (abcde)*	71.2	62.7	78.8	n.d.	-	-
v43	*GLU_c (abcde) → GLU_m (abcde)*	74.8	66.4	82.5	0	0	0
v44	*GLU_m (abcde) ↔ AKG_m (abcde)*	161.0	158.5	179.8	n.d.	-	-
v45	*GLU_c (abcde) ↔ GLU_{ex} (abcde)*	6.3	3.4	9.3	1.3	1.2	1.6
v46	*GLU_c (abcde) → Biomass*	9.7	-	-	0	-	-
v47	*GLN_c (abcde) → GLU_c (abcde)*	14.6	13.3	15.8	0	0	0
v48	*GLN_c (abcde) → GLU_m (abcde)*	81.0	80.1	82.4	0	0	0
v49	*GLN_{ex} (abcde) ↔ GLN_c (abcde)*	66.4	50.3	82.4	1.28	1.02	1.78
v50	*CIT_c (abcdef) → OAA_c (fcde) + $AcoA_c$ (ab)*	39.1	-	-	0	-	-
v51	*$AcoA_c$ (ab) → Biomass*	39.1	-	-	0	-	-
v52	*AA_{ex} (ILE, VAL, MET, THR, TYR, PHE) → MAL_m (AA catabolism)*	49.6	5.1	99.9	0	-	-
v53	*AAex (LEU, ILE, LYS, TYR, PHE) → $ACOA_m$ (AA catabolism)*	92.6	27.9	161.4	0	-	-
v54	*AKG_c (abcde) → GLU_c (abcde) (AA catabolism)*	38.7	5.2	75.6	0	-	-
v55	*AA(PRO, HIS, ARG) → GLU_c (AA catabolism)*	11.6	0.8	25.3	0	-	-
v56	*PYR_{c1} (abcd) ↔ ALA_c (abcd)*	0	-	-	0	-	-
v57	*PYR_m (abc) ↔ LAC (abc)*	0	-	-	0	-	-
v58	*PYR_{c1} (abc) ↔ PYR_{c2} (abc)*	168.1	149.2	174.8	0.13	0	0.41
v59	*PEP (abc) → PYR_{c2} (abc)*	122.7	103.8	129.4	0	0	0
v60	*G6P (abcdef) → carbohydrates*	8.9	-	-	0	0	0
