# Supplementary figures and images for: Non-stationary 13C metabolic flux analysis of Chinese hamster ovary cells in batch culture using extracellular labeling highlights metabolic reversibility and compartmentation
Source: BMC Syst Biol. 2014 Apr 28;8:50. doi: 10.1186/1752-0509-8-50 (PMC4022241; doi:10.1186/1752-0509-8-50)

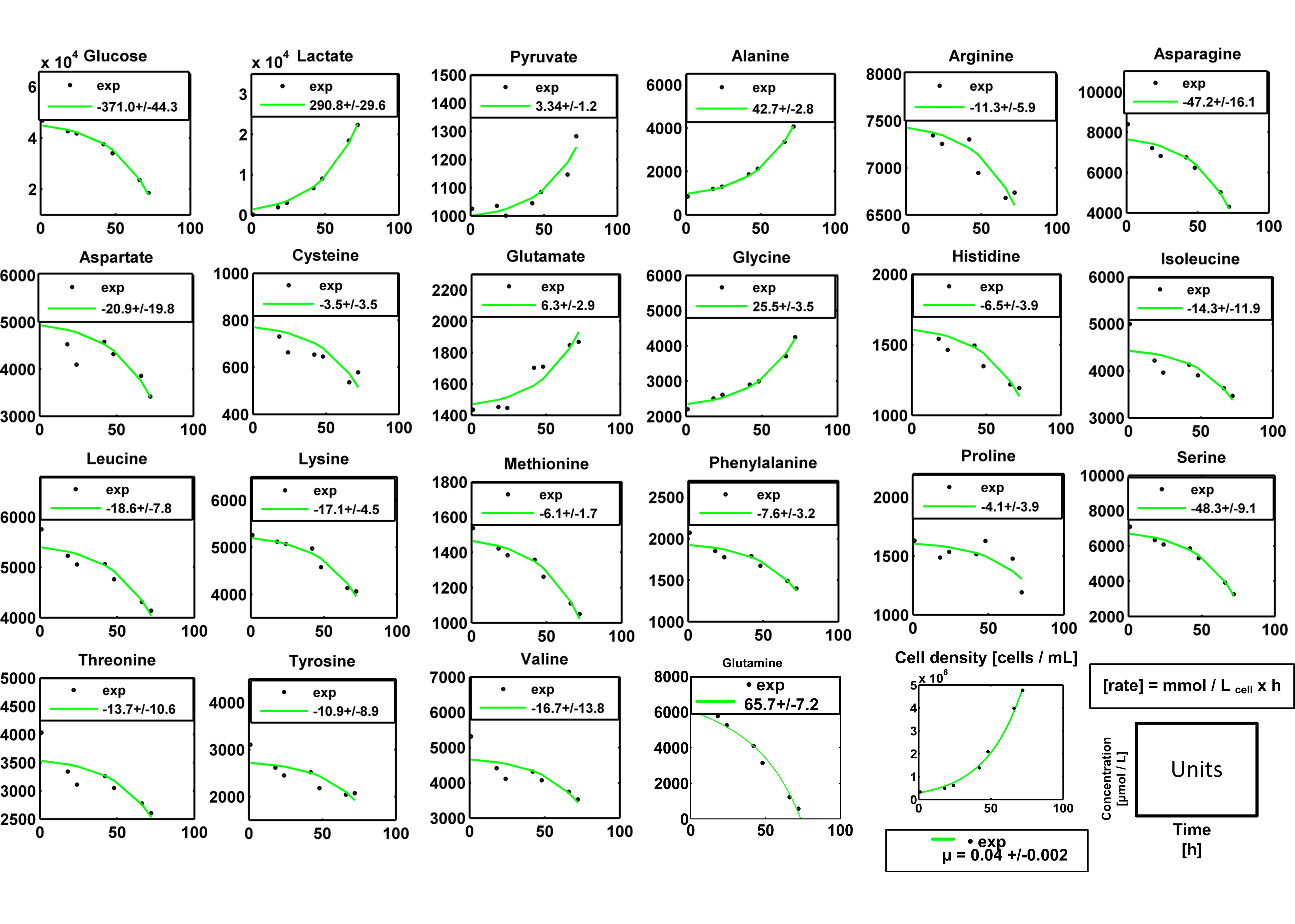

Supplement: Additional file 2 — Complete culture profile of CHO-K1 during the exponential growth phase.The lines represent the fitted concentration profiles to the experimental values (dots) and in the boxes are the determined extracellular rates [mmol/(L cell × h )] together with the 95% confidence intervals. Glutamine uptake was determined by considering a spontaneous degradation rate of 0.0033 h−1. The exponential growth phase is shown in the last plot. [file 1752-0509-8-50-S2.tiff]
